# Herbal medicine use during pregnancy and childbirth: perceptions of women living in Lilongwe rural, Malawi – a qualitative study

**DOI:** 10.1186/s12905-023-02387-z

**Published:** 2023-05-04

**Authors:** Dziwenji Makombe, Enalla Thombozi, Winnie Chilemba, Alexander Mboma, Kondwani Joseph Banda, Elias Mwakilama

**Affiliations:** 1 Department of Community Health Nursing, Kamuzu University of Health Sciences, Lilongwe, Malawi; 2 Department of Midwifery, Kamuzu University of Health Sciences, Lilongwe, Malawi; 3 College of Nursing, School of Nursing, Taipei Medical University, Taipei, Taiwan; 4 Endoscopy Unit, Department of Surgery, Kamuzu Central Hospital, Lilongwe, Malawi; 5 Department of Mathematical Sciences, University of Malawi, Zomba, Malawi

**Keywords:** Herbal medicine, Pregnancy, Labour, Perceptions, Rural Malawi

## Abstract

**Background:**

Globally, use of herbal medicine during pregnancy and labour is often associated with adverse obstetric outcomes such as uterine rupture and fetal distress. However, in rural Malawi, information on the perceptions of women about the use of herbal medicine during pregnancy and labour is underreported despite the practice. Understanding women’s views and perceptions on use of herbal medicine during pregnancy and labour is therefore critical for understanding the basis of their practice and for setting up maternal and neonatal health care interventions to alleviate any possible pregnancy and labour complications.

**Aims:**

To explore the perceptions of women on the use of herbal medicine during pregnancy and labour in rural Malawi.

**Methods:**

We employed a qualitative descriptive (QD) study on the purposively identified participants (women with parity $\ge 2$), residing in four villages (Kagona, Champsinja, Mthupi and Manja) of Traditional Authority Malili, in Lilongwe rural district, Malawi. Qualitative data was collected through four Focus Groups of 6–8 women in each group that were conducted in each village. Data analysis was performed inductively, using reflexive thematic analysis approach.

**Results:**

A total of 28 women of reproductive age 20 and above; 20–24 (32.14%), married (75%), average of 3 deliveries (57.14%), primary school education (75.0%), and Christians (92.86%) were recruited and interviewed. Two main themes emerged from the narratives: (1) perceived benefits of using herbal medicine: (i) hastens labour, (ii) prevents pregnancy complications and (iii) prevents and treats illnesses, and (2) perceived risks of using herbal medicine: (i) perceived maternal risks, (ii) perceived fetal risks.

**Conclusion:**

In rural Malawi, the practice of using herbal medicine during pregnancy and labour is perceived as both risky and beneficial to women. These perceptions are shaped by the exposure to either personal or other people’s experiences, hence the continued practice. Therefore, inclusion of health education topics on maternal complications due to use of herbal medicine among women can help reduce maternal and neonatal mortality rates in rural Malawi. Further research is also warranted to explore accessibility and community pathway systems for herbal medicine use during pregnancy and labour among the pregnant women.

**Supplementary Information:**

The online version contains supplementary material available at 10.1186/s12905-023-02387-z.

## Background

Globally, use of herbal medicine during pregnancy and labour among women is estimated at 60% in the western countries [1–5] and between 34 and 80% in the Sub-Saharan Africa (SSA) [6–8]. The proportional differences are attributed to existing variations in cultural practices, economic status, and access to conventional medicines and health care services [9]. Often times, women use various forms of herbal preparations such as parts of plants, plant materials or combinations thereof during pregnancy and labour [10]. Thus, understanding women’s reasons for using herbal medicine during pregnancy and labour can help identify approaches to address the existing access to health service challenges, thereby improving maternal and neonatal health outcomes.

The prevalence of herbal medicine use in pregnancy and labour in Malawi was estimated at 25.7% in 2018 [11]. The common herbal medicine considered for inducing and hastening labour is “*Mwanamphepo”* (cissus/Vitaceae plants species) [12, 13]. This herbal drug is divided into four categories, depending on its use; (i) *Mwanamphepo wa Magazi* (Ampelocissus obtusata), (ii) *Mwanamphepo Woning’ina*, (epiphytic), (iii) *Mwanamphepo wa Mtawaleza (cissus aristolochiifolia)* and (iv) *Mwanamphepo wa Mng’ono.* The first three are primarily employed to hasten labour, while the last type of herb is used for treating pregnancy related illness such as nauseas, vomiting and lower back pain [12]. Previous study findings reveal that herbal medicine is associated with maternal complications because its use during labour induces strong continuous contractions that do not correspond to the slow dilatation of the cervix [14]. As such, use of herbal medicine has been linked with adverse obstetric outcomes including uterine rupture and fetal distress [15], contributing to increased maternal and neonatal mortality rates [11]. Surprisingly, some women continue to use herbal medicine during pregnancy and labour in the presence of easily accessible health care services in Malawi [16], for some unknown reasons. Consequently, a majority of these women are at an increased risk of several adverse health outcomes including obstetric complications which lead to an increase in maternal and neonatal mortality rates [17].

Current evidence demonstrates variation on women’s perceptions on use of herbal medicine during pregnancy with some authors arguing that herbal medicine is more effective, more accessible, and safer for the fetus than conventional medicine and may be used to treat illnesses in pregnancy and improve fetal wellbeing [18]. In light of this evidence, use of herbal medicine has shown to facilitate labour and treat common illnesses during pregnancy and labour including nausea and vomiting, urinary tract infections, common cold/flu, and also preventing potential miscarriage cases [19–21]. On the contrary, facilitation of labour using herbal medicine has been associated with bad obstetric outcomes including uterine rupture and fetal distress [22]. Similarly, few studies conducted in Malawi have reported that some herbal medicines which are believed to hasten labour have oxytocic properties that cause obstetric complications [15]. Moreover, results from a systematic review revealed that use of raspberry leaf to induce and shorten labour is associated with fetal distress, leading to cesarean section [23]. Furthermore, ingestion of castor oil for induction of labour has also been associated with an increase in meconium-stained liquor indicating fetal distress leading to caesarean deliveries [24]. Therefore, owing to the current research gap, we conducted a qualitative descriptive study to explore and understand the perceptions of rural women on use of herbal medicine during pregnancy and labour in rural Malawi. The study has a potential to contribute to the body of empirical evidence and reduce martenal and neonatal mortality in the country.

## Theoretical framing – health belief model

**Fig. 1:**
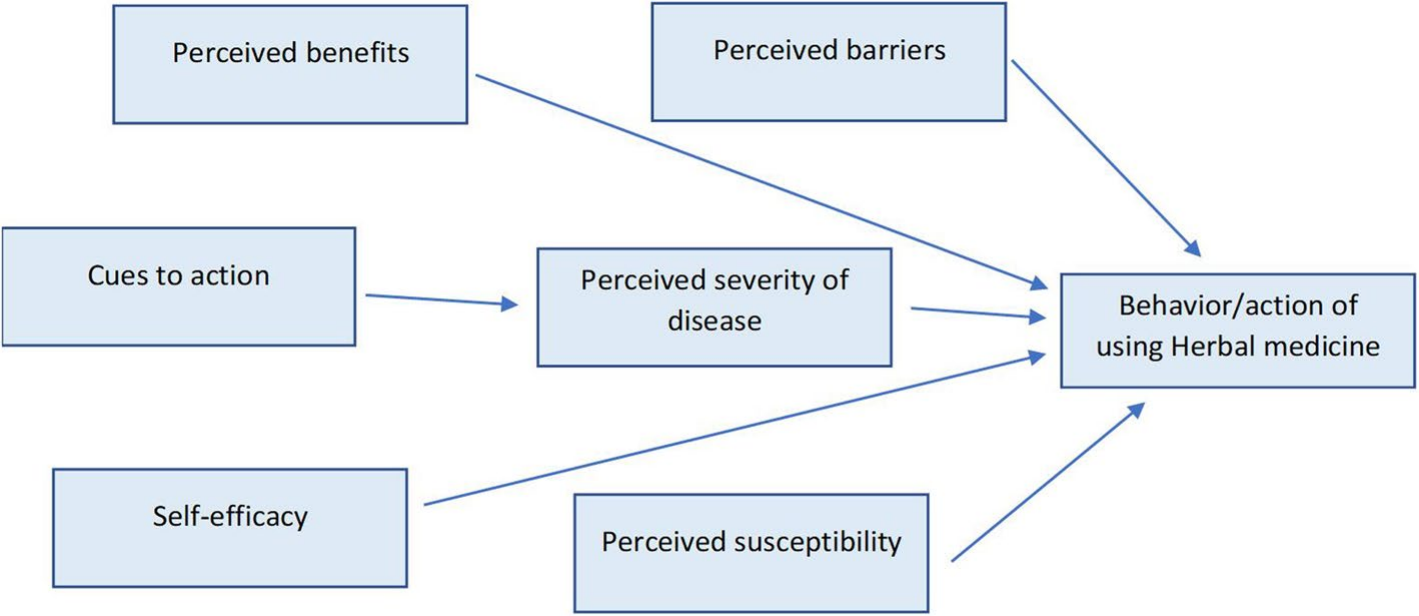
Theoretical framework of Health Belief Model as applied to Herbal Medicine Use among Women in rural Malawi study

Over and above the factors that influence adoption of certain practices on the basis of perceptions, this study considered a holistic approach to understanding the experiences that rural women in Malawi have during pregnancy and labour. Thus, this study uses lens of concepts drawn from the Health Belief Model (HBM) to research on the perceptions or views for herbal medicine use (Fig. 1). Proposed by Rosen-stock and his colleagues in 1950s [25], and the HBM focuses on predicting health behaviors and explaining why few people would participate in programs to prevent and detect disease [26]. Particularly, the HBM is applied to reveal what encourages or discourages people from participating in disease prevention activities on the basis that people’s beliefs about whether they are susceptible to disease or not, and their perceptions of the benefits of trying to avoid it, are influenced by their readiness to act [27]. The HBM hence focuses on assessing health behaviours of individuals through examination of perceptions and attitudes that someone may have towards disease and negative outcomes of certain action.

The HBM proponents that there are six main constructs that influence people’s decisions about whether to take action to prevent a disease, screen for or control illness, namely; (i) Perceived Susceptibility which explains a belief that one will either or not contract a disease. Perceived susceptibility ranges from being afraid of contracting a disease to completely denying that certain behaviors will result in illness. In the context of this study, women would use herbal medicine to hasten labour with a belief that the known complications cannot happen to them (they are immune), (ii) Perceived Severity of disease which explains one’s opinion on how serious a condition and its consequences are. This component is related to how much the person knows about a disease and the knowledge can influence either behavior. For instance, if a woman has knowledge that use of herbal medicine during labour is associated with ruptured uterus and fetal distress she is very unlikely to use the herbal medicine, (iii) Perceived Benefits which explains the belief that taking action (preventive measure) would reduce susceptibility to the condition or seriousness of impact. In the study, it is applied that women would stop taking herbal medicine during pregnancy and labour when they perceive that it is of benefit i.e. prevents ruptured uterus and post partum hemorrhage which may lead to death, (iv) Perceived Barriers which explains that people can take action if they believe that the cost of taking action are outweighed by the benefits. For instance, women may consider the prevention of herbal medicine use in pregnancy if they see that it is not that difficult to wait for normal delivery and it is of more benefit than the associated harm, (v) Cues to Action which explains that people can only act positively if they have ever been exposed to factors that prompt action. For instance an experience of ruptured uterus or still birth following use of herbal medicine would directly inform the woman’s perceptions on herbal medicine and vice versa. Health education from health workers, the media, family and friends are other forms of cues to influence action, and (vi)Self-Efficacy which explains the instance whereby an individual feels confident that can take action to achieve the desired outcome. For instance, it is only when the women understand that they are susceptible to the severity of dangers of using herbal medicine (can have ruptured uterus, postpartum hemorrhage), they will develop the confidence to refrain from using it hence practice healthy behaviours.

This study is therefore guided by the building blocks of the HBM theory (Fig. 1) by providing insights on the existing perceptions of women on herbal medicine use during pregnancy and labour. Additionally, the theory assisted the researchers to establish why women continue to use herbal medicine in pregnancy despite the existing prevention messages in the media and different cues to action. The discussion section is also guided by the components of the theory and interpretation of the findings as connected to the focus of the theory, allowing the researchers to make recommendations on how the use of herbal medicine practice could be handled in communities to reduce maternal morbidity and mortality rates in rural Malawi.

## Methods

### Study design and setting

This study employed a qualitative descriptive (QD) study design approach. The QD minimizes the chances of verbal misrepresentations which often arise during field data transformation, since in the QD design, researchers stay closer to their data and to the surface of words and events than in ethnographic or narrative study designs [28]. The QD design, moves beyond literal description of data and attempts to interpret the findings without moving too far from that literal description [29]. In addition, the QD design permits a researcher to transform the data and provide answers to research questions of special relevance to practitioners and policy makers [28]. Understanding perceptions of women on herbal medicine use during pregnancy and labour is of special relevance to midwifery practitioners and has the potential to inform policy makers. In addition, the study was conducted within the community setting to create a natural environment as much as required by a qualitative design [30], which allows the phenomena being explored to present itself as if it were not under study [28]. The study was conducted in 4 villages of Kagona, Champsinja, Mthupi and Manja under Traditional Authority (TA) Malili, in Lilongwe rural in Malawi. These villages are located 5 Kilometers from the mid-south of Likuni community in Lilongwe City, indicating that residents from these villages including women have access to health care services from two nearby hospitals, Bwaila and Likuni. In addition, these villages were selected following a community diagnosis activity conducted by a previous study revealing that out of the 101 study respondents, 26% admitted ever using some herbal medicine during pregnancy and labour [31].

### Recruitment and sampling

Study participants were adult women (of at least 18 years at the time of the study) purposively sampled into the study. These were women who had ever experienced at least two deliveries (parity $\ge$ 2), selected to allow them share perceptions from their self-reported lived and observed experiences. To enrich the study findings and elicit a diversity of opinions, a maximum variation of purposively sampled population was employed. Thus, women of different age categories, despite setting minimum study age to 18 years; different faith groups; and different level of education were recruited [32, 33]. The criteria ended up identifying 28 women from all the sampled villages.

### Ethical consideration

Ethical approval for the study was obtained from the College of Medicine Research and Ethics Committee (COMREC) of the University of Malawi (reference number P.10/20/3169). In addition, informed consent was obtained from the study participants prior to their participation in the study’s focus group discussions (FDGs) and were at liberty to withdraw from the study at any time. Participant identifiers, and not names, were therefore used during data manipulation and analysis to maintain privacy and promote anonymity.

### Data collection

Data was collected using FDGs through administering a semi-structured interview guide (Additional file 1: FGD guide_English) between 21^st^ to 30^th^ December, 2020. The guide was designed to elicit information on their perceptions regarding herbal medicine use during pregnancy and labour. The use of a semi-structured interview guide allowed women to freely express their feelings and perceptions on the questions presented. In addition to demographic information, some questions included information on their sources of information about herbal medicine, experiences and perceptions, perceived benefits or risks, and factors that influence women to use herbal medicine during pregnancy and labour. Women were encouraged to share their individual experiences or from a general knowledge, but particulary the former case. Four FGDs were held with 7 participants in each group. The FGD method of collecting qualitative data was chosen because studies have indicated that women prefer to discuss matters related to use of herbal medicine face-to-face and thus, not to be considered as deviants to health care protocols [34] and also data collection using a FGD guide allows researchers to get collective views, mostly perceived by all the participants regarding a phenomenon.

The interviews were conducted in Chichewa; a local language used in the area of the study, and were audio-recorded with participant’s consent. To ensure quality of the collected data, the developed interview guide was first pretested with a FGD involving seven women in Chimwanya village under TA Chiputula after seeking permission from the Lilongwe district council. This aimed at rating the length of time taken to conduct data collection; identifying questions that were not clear or misinterpreted, determining the sequence of questions and ensuring that the tape recorder was working satisfactorily [35, 36]. Evaluation of this activity indicated that the study tools were all appropriate for the data collection process.

To familiarize with the interview guide, the researchers read the interview guide several times. The FGDs were all conducted in a quiet environment where audio privacy was greatly observed. The FGD notes collected during the facilitated discussions were then reviewed by DM, a nurse and midwife who had received prior training from ACEPHEM in qualitative methods, alongside another member of the research team, AM, at the end of each discussion for a reflection purpose and a way of analyzing the emerging data. All the recorded interviews were kept in a laptop, secured with a password, in preparation for the analysis. On average, each FGD took approximately 40 to 60 min.

### Data analysis

Study demographics data were descriptively analysed in Microsoft Excel software, and verified by EM. The authors listened to the audio files and then transcribed them verbatim. They were then translated to English by an experienced secondary school English teacher, and checked for accuracy by DM, AM and JB. The qualitative data was analyzed manually using reflexive thematic analysis which allows subjective interpretation of themes of text data through a systematic classification process of coding and identifying themes or patterns [37]. In this study, codes were generated from the responses that addressed research questions and referred to the theoretical framework that underpinned the study (Table 1). This procedure was inductively done to allow the researcher use the categories and names for categories to flow from the data, rather than using preconceived categories [37, 38]. The following steps, as articulated by Heish and Shannon [37], were utilized in the analysis of the data. Step 1: Becoming familiar with data: The authors DM, WC and ET began the analysis by reading through the data repeatedly to be familiar with the content. Step 2. Coding process: Then the author DM and AM read word by word to derive codes by highlighting texts that manifest to describe perceptions and write in the margins of the text a keyword or phrase that meant to capture the perception, using the participant’s words (See Table 1). Step 3. Develop Categories: The authors, DM and WC developed categories as headings from the developed codes and grouped them according to their related content. Step 4. Thematic development: Finally, the authors developed themes by summarizing the developed codes into single defining sentences called “themes”. Two themes were developed from the data namely, (a) Perceived benefits of herbal medicine in pregnancy and labour; and (b) Perceived risks of using herbal medicine in pregnancy and labour.

**Table 1:**
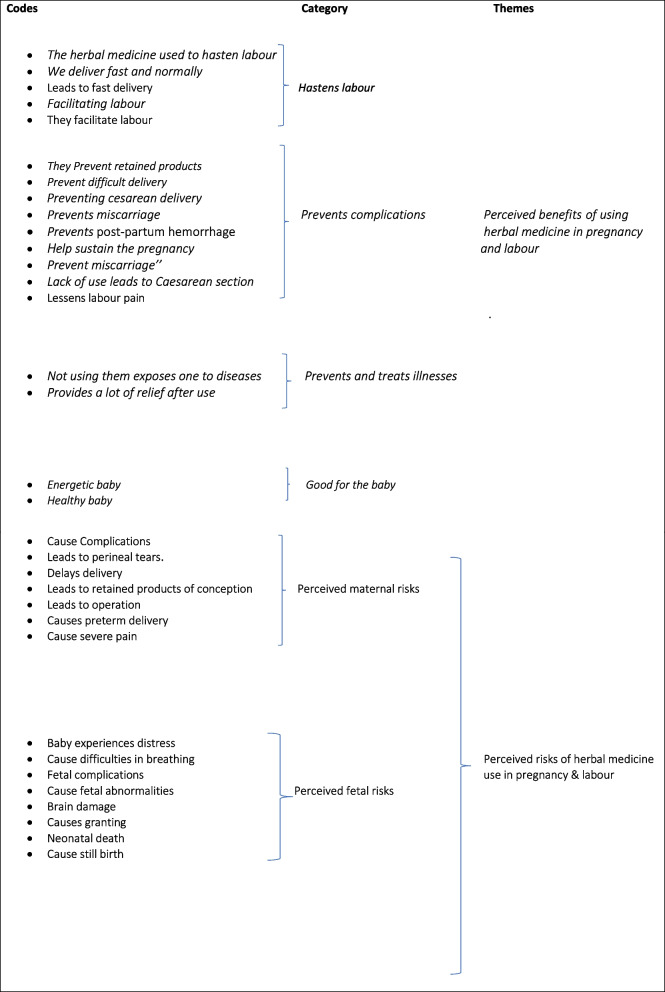
Data analysis- key codes and key themes

## Results

### Socio-demographic characteristics of study participants

A total of twenty-eight women were recruited and interviewed in this study. The majority were between the age of 20–24 (*n* = 9, 32.14%), and 30–34 (*n* = 9, 32.14%). Of these women, 75% were married, and 57.14% had an average of 3 deliveries (parity 2–3) in their life. Furthermore, out of the 28 participants, 21 (75.0%) indicated to have ever attended primary school at the time of the survey. In terms of religion, the largest number of the participants were Christians (*n* = 26, 92.86%) and only two were Muslims (7.14%) (Table 2).

**Table 2:** Socio-demographic characteristics of study participants (*N* = 28)

Variable	Numbers of Participants (%)
**Age**	
20–24	9 (32.14)
25–29	9 (32.14)
30–34	6 (21.43)
35–39	3 (10.71)
40 and above	1 (3.58)
**Marital status**	
Married	21 (75.0)
Divorced	3 (10.71)
Widowed	3 (10.71)
Single	1 (3.57)
**Deliveries (parity)**	
2–3	16 (57.14)
4–5	10 (35.71)
6 and above	2 (7.15)
**Religious affiliation**	
Christianity	26 (92.86)
Islam	2 (7.14)
**Highest education level**	
None	1 (3.57)
Primary	21 (75.0)
Secondary	6 (21.43)

### Themes

Narratively, qualitative results were grouped into two major themes. Each theme has been presented with its respective categories.

### Percieved benefits of using herbal medicine during pregnancy and labor

The participants perceived the use of herbal medicine during pregnancy and labour differently. While some participants presented positive perceptions, others felt use of herbal medicine during pregnancy and labour had negative effects towards health of the woman or the baby.

#### Herbal medicine hastens labour

Several participants perceived that use of herbal medicine during pregnancy and labour facilitated the labour process. The following sentiment demonstrates this:

• P.5 “Women take the herbal medicines so that they deliver fast when labor starts”.

• P.7 “Yes. when you take herbal medicine you deliver fast, it works faster for you so that you rest from labour”.

• P.3 “Mmmh, when you are pregnant, many people give you different suggestions some say that it is important that you take herbal medicine that you deliver fast when labor start”.

• P.2 “Indeed some say you should Immersing leaves of katate or roots of chewe okra and drinking its concoctions is one of the herbal medicine used to hasten labour that it is taken as soon as labour starts’’.

[All participants nodded] (FGD 1).

Additionally, most participants’ narratives described their dependence on herbal medicine use during pregnancy. For example, some participants said;

• P.1 ‘’We as village people really depend on herbal medicine because we see that when we take herbal medicine we deliver fast and normally without problems’’**.**

• P.6 “But one time I encountered something when I was at the hospital. A woman took herbal medicine so that she delivers fast”.

• P.3 “But it really helped me because I could get the pains on and off here at home but when I took the herbal medicine…I delivered immediately I got to the hospital”.

• P.4 “I took tea leaves and roots of katate okra during my third pregnancy….As soon as I felt labour pains …..when I got there, I immediately delivered”.

[All participants nodded] (FGD 2).

### Herbal medicine prevents pregnancy complications

Several of participants' narratives indicated that using herbal medicine during pregnancy and labour prevented them from undergoing cesarean delivery and hence having a spontaneous vaginal delivery. For example, they narrated that the use of herbal medicine such as “*mwanamphepo*” during pregnancy prevents them from having a caesarean delivery.

The following sentiments indicate:

• P.1 “But when I left my home village to live here, I ended up having an operation because I never used the Mwanamphepo herbal medicine”.

• P.2 ‘’yes…I take Mwanamphepo herbal medicine every time I go into labour, this helps in facilitating labour and preventing cesarean delivery…”

• P.5 “Very true, as for me, I feel that not taking herbal medicine in my recent pregnancy exposed me to mmmh cesarean section …therefore the herbal medicine really prevents one from undergoing cesarean section”.

[Many participants nodded] (FGD 2).

Similarly, participants from the other groups indicated same sentiments. For instance:

• P.1 “Mostly because these days whenever a women fails for a short time to push…she is taken to theatre for an operation…..you see….so all that is influencing people to think of using herbal medicine”.

• P.2 “And also it happens that you had a Caesarean section in your first pregnancy…and when you get the second pregnancy…you take herbal medicine so that you shouldn’t have caesarean section”.

[Most participants nodded] (FGD 4).

Most commonly, participants’ narratives describing the beneficial aspect of herbal medicine use during pregnancy and labour indicated a perception that herbal medicine sustains pregnancy and prevents miscarriage and pregnancy complications such as post-partum hemorrhage. Participants described beliefs that if a husband had multiple sexual partners when his wife was pregnant, the probability of *blood mix from the other women* was very high causing postpartum bleeding as soon as the woman had a delivery. Hence, to prevent this complication, women took herbal medicine so that they remained safe during delivery***.***

[All participants nodded] (FGD1).

Similarly, some participants believed that immersing dry groundnuts and then drinking its boiled solution was another type of herbal medicine, which prevents miscarriage. The participants said;

• P.2 “The other herbal medicine that helps in sustaining pregnancy looks like okra….but it is put in porridge to prevent pregnancy loss”.

• P.1 “Aaah yes, I have ever experienced miscarriage. I was given herbal medicines to be drinking and some to be taking through porridge and I never experienced pregnancy loss again”.

• P.1 “Myself, when others get pregnancy… and experience consecutive ….miscarriage, they go to collect herbal medicine….so that they should not miscarry….so it really helps and they really don’t miscarry the pregnancy”.

[All participants nodded]- (FGD 4).

### Herbal medicine prevents and treats pregnancy-related illnesses

Most participants also reported that use of herbal medicine during pregnancy prevents frequent illnesses due to its curative properties. In their statements, it is stated that if one has not used herbal medicine, she suffers a lot of illnesses than those that have used before. These findings are narrated as follows:

• P.2 “The other benefit of using herbal medicine is that, when you are pregnant, you are prone to illnesses and the like…. For example, I used to experience severe leg pain…but when I used that herbal medicine I really felt relieved a lot**”….**

• P.5 “Not using them exposes one to diseases and even ends up having cesarean delivery because of lack of protection from illnesses’’

[All particpants nodded]- (FGD 2).

Similarly, Participants in FGD 3 had the following views;

• P.5 “In my third pregnancy, I had some problems with my appetite so I took herbal medicine and my appetite improved”.

• P.4 “True aaah.. mmh…some experience leg pain during pregnancy so when they take herbal medicine they feel a lot better”.

• P.6 “Eeeh…when you go to antenatal clinic and you are told that the blood is not enough in your body we are advised by the nurse to boil papaya leaves and drink its concoctions….or CHIKHADA and then drink its concoction for the benefit of your body”.

### Herbal medicine is good for the baby

A few women also expressed that herbal medicine is not only taken for their benefit; but also for the unborn baby to be energetic when she/he will be born. The following sentiments illustrate:

• P.7 “Whilst during pregnancy you take the herbal medicine so that you should not have difficulties in the future but the unborn baby should be energetic”.

[A few Participants agreed] (FGD 1).

### Percieved risks of using herbal medicine during pregnancy and labour

Some of the participants perceived herbal medicine use during pregnancy and labour as risky behaviour. The narrations of these participants indicated that the intention to take the herbal medicine was good but the results were undesirable.

### Percieved maternal risks

Cesarean delivery was one of perceived risks or consequence of using herbal medicine during pregnancy and labour. Narratives indicated a perceived link between cesarean delivery and herbal medicine use. Most commonly, participants from all groups were able to share perceptions derived from their encounters. Many participants indicated that the intention of taking the herbal medicine was to facilitate labour but ended up having a caesarian delivery. The following statements suggest:

• P.5 ‘‘I took juice extract from leaves of Katate okra when my labour started to hasten it. However, this did not go according to expectations as labour couldn’t progress well until I was taken to theatre for Cesarean delivery so I felt it did not work for me, herbal medicine use led to the operation’’.

• P.7 “True…mmmh (laughs) as for me I ended up being operated on… They gave me herbal medicine to be taking… tea leaves and katate”.

• P.4 “Me too…(laughs)..I took tea leaves and roots of katate okra during my third pregnancy to quicken labour…but aaah they caused me to end into theatre for ceasarean section”.

• P.1 “As for me I really experienced the dangers of using herbal medicine in pregnancy…because I underwent cesarean section”.

• P.3 “As for me I would say that I wouldn’t use them anymore because …I ended up going for an operation because of the same herbal medicine”.

[Majority of participants nodded] (FGD 2).

In addition to the perception that herbal medicine used during pregnancy and labour can lead to cesarean delivery, in another group it was mentioned that it would also bring out a complication in the womb. Uterine rupture and maternal death were specifically mentioned in the following quote:

*“I just want to add that herbal medicine is really bad because when you take that medicine you can even bring out a complication on a womb to the point of being operated on because the time that the baby was supposed to be born is when other people were saying take the herbal medicine and at the same time you end up with complications, the baby is born with full force …and also it may cause uterine rupture…. Sometimes the woman can even die because of the herbal medicines taken”. [Agreed by all participants] (FGD 1).*

Delivering on the way to the hospital after taking the herbal medicine is another risk that was shared by the participants. The following results were from personal experiences: A good number of participants shared their personal experience indicating that:

P.2 “*The problem with herbal medicine, there is a problem which comes in if you have taken herbal medicine when labour has started.,,and then you leave for the hospital…you end up having your delivery on transit because of the herbal medicine”.*

P.4 “*Aaah that’s very true herbal medicine is somehow not beneficial because you really deliver on transit to the hospital…because I have ever delivered in transit…..I couldn’t reach the hospital after taking herbal medicine”.*

P.5 “*Mmmh ofcourse the benefit of herbal medicine is that you experience a fast delivery…but it is very risky because you intend to delivery as soon as you get to the hospital but you don’t even make it to the hospital”, it happened to me”.*

[All participants nodded] (FGD4).

### Percieved risks to the fetus

Most commonly, participants did not only perceive herbal medicine use during pregnancy as risky in the view of a woman but also to the unborn baby. The following sentiments demonstrate this:

• P.1”The baby could be born with severe difficulties in breathing, or even convulsions because of the herbal medicine that the woman took”.

• P.2 “It is really not good for a pregnant woman to take herbal medicines, not good to the unborn baby”.

• P.3 “I agree with what they are saying here that the baby can be born with difficulties in breathing”.

• P.4 “Even sometimes maybe the baby was just okay but now it will be born with abnormalities, or even sometimes the baby’s eyes can bel”

• P.5 “You really take the herbal medicines whilst when you deliver the baby you find out that the baby has severe difficulties in breathing, others indeed end up delivering a still birth”.

• P7. “Mmmhaaah its indeed true.herbal medicine is really bad mmh sometimes you can give birth to a baby with abnormalities who cannot walk”.

[All participants nodded] (FGD 1).

## Discussion

This study explored perceptions of women on the use of herbal medicine during pregnancy and labour in rural Malawi. The participants were women who had given birth at least twice in their reproductive period. Two themes emerged from the narratives: (1) Perceived as beneficial: (i) hastens labour, (ii) prevents pregnancy complications, (iii) prevents and treats illneses, and (2) perceived as risky practice**:** (i) Percieved maternal risks, (ii) perceived fetal risks.

### Perceived benefits on the use of herbal medicine

We found that women’s perceptions to use herbal medicine in rural Malawi; Somewhat lean towards benefits and risks. Benefits of use included hastening labour; prevented pregnancy complications and illnesses. C*hewe* okral solution, was identified to be effective in hastening labour. Similar findings are presented by studies done in Zambia and Malawi where “bush okral” and “Mwanamphepo” were mentioned respectively [12, 39]. It can be presumed that Chewe okra contains oxytocic properties as Mwanamphepo. The fact that the dosage in these herbal medicines is not controlled attests to the risk of uterine rupture and fetal distress due to overdose of oxytocin. Therefore, consistent with other researchers, evidence on properties of Chewe okra and establishment of herbal medicine monitoring policies might help reduce these complications [40]. Furthermore, women in this study used herbal medicine to quicken labour hence escape ceaseran delivery. Most women added that doctors rush them to theatre instead of giving them more time to experience normal labour. Similar findings are presented in a study done in Nigeria [41]. It can be recommended that information giving to clients should be encouraged so that they are well informed of the reasons the decision to undergo ceasaren delivery is taken.

Prevention of postpartum hemorrhage was another perceived benefit of using herbal medicine. Participants alluded that lack of use is risky especially among women with infidel partners because a woman could even bleed to death after delivery. A study conducted in Zambia reported similar findings and stated that women use herbal medicine to prevent obstetric complications when their spouse has infidelity issues [39]. Arguably, Peprah et al. [9] allude that use of herbal medicine is somewhat cultural related and this finding attests that culturally, women use herbal medicines during pregnancy in the context of belief. Thus, health education on herbal medicine use during pregnancy and labour should be encouraged in practice.

Furthermore, the study revealed that women used herbal medicine to prevent abortion. Kabuluzi [17], and Maliwichi-Nyirenda and Maliwichi [42] reflect similar findings in studies done in Malawi. However participants in this study mentioned ingestion of groundnuts solution, while in the Maliwichi-Nyirenda and Maliwichi’s [42] study the herbal medicines were tied in the woman’s waist. This difference could be attributed to cultural beliefs [42, 43]. Nevertheless, it indicates that women have a variety of herbal medicine they use during pregnancy depending on their needs at a particular time. Protection from frequent illnesses and treatment of leg pain, pelvic infections and nausea was the other perceived benefit. This finding is consistent with Illamola et al. [44] and a multi-national study done in almost 17 western countries including Australia, the United Kingdom, and United States of America (USA), where ginger and cranberry were used by pregnant women to treat illnesses [21]. Much as the current study finds that herbal medicines may achieve successful cure of illness, evidence of safety cannot be guaranteed in many African countries including Malawi. This is attributed to the fact that in western countries, herbal medicines are legalized and controlled. Hence, they are provided at the health facility and pregnant mothers are monitored by clinical staff hence potential adverse events are easily averted [8, 16, 45].

### Perceived risks of using herbal medicine

Despite the study revealing that women would perceive the use of herbal medicine as beneficial, some participants perceived it as a risk. Ceasarean delivery, ruptured uterus and death, on-transit delivery and fetal distress and stillbirth were the perceived risks mentioned by the participants. In their sentiments, they indicated that in the process of taking herbal medicine to hasten labour, a complication such as fetal distress rises and the decision to deliver through cesarean section is taken. This finding is consistent with results by Mkize [7] who revealed that herbal medicines used to hasten labour leads to strong, continuous contractions not corresponding to the slow dilatation of the cervix, causing meconium stained liquor which ends in caesarean section [7, 23, 24, 34]. This consistency is evident because scientifically, when synthetic oxytocin is used to hasten labour, contractions and fetal heart are monitored closely. The drug is stopped if it alarms that it may bring out complications or not produce the desired outcome [46]. Nevertheless, this is not the case when women choose to use herbal medicine to hasten labour since the dose is not regulated and parameters of maternal fetal monitoring are not considered risking complications such as ruptured uterus and fetal distress.

Furthermore, in the present study, women revealed that the use of herbal medicine during labour may lead to “womb complications” such as ruptured uterus and even death. These findings are consisted with those of Zamawe et al. [11], Mukasa et al. [47], and Lampiao [48] which highlighted ruptured uterus and fetal distress as complications associated with hebal medicine use during pregnancy. This finding, therefore, provides a basis to health workforce that the public already has some knowledge of the risks of herbal medicine use during pregnancy and labour. On-transit to delivery was also highlighted as the other risk. Participants specified that the medicine is taken to hasten labour but sometimes they fail to make it to the health facility and deliver on the way. Although, there is limited literature to support such claims, the present study finding is of great significance. Arguably, scientific evidence from one of the studies assessing properties in the herbal medicine commonly used to hasten labour in Malawi alludes that it has oxytocic properties [15]. This is so because oxytocin aid in contractions power hence delivery may be precipitated.

Finally, the current study established that stillbirth and severe birth asphyxia that demands resuscitation with oxygen are some of the perceived risks of using herbal medicine during pregnancy and labour. Participants indicated that some herbal medicine risks the health of unborn baby leading to difficulties in breathing and even stillbirth. Consistently, increased uterine activity, featl distress and hypoxia were also mentioned as risks in studies done in Malawi and South Africa respectively [11, 49]. A study conducted in Uganda claimed that women who use herbal medicine during labour are four times likely to give birth to a neonate with a low APGAR score [50], hence indicating asphyxia. A literature review done on safety of herbal consumption during pregnancy validates similar findings by indicating that use of herbal medicine during pregnancy have the adverse effects that can actively harm the fetus [51].

### Strengths and limitations

The study’s diverse views from women of different age groups and parity present its major strength. However, considering the breath of the study topic, the study’s purposive limited sample size and use of FGDs alone, somehow limit transferability of the findings. Nevertheless, this study has improved the qualitative empirical evidence regarding the use of herbal medicine during pregnancy and labour in rural Malawi. It has not only answered the question of ‘’why would women decide to use or not use herbal medicine during pregnancy and labour’’, but also identified herbs used during pregnancy and labour. These herbals can then be scientifically embraced to establish their effects hence inform the health workforce and policy makers of their potential use in the health systems delivery in Malawi.

## Conclusions and future directions

Using herbal medicine, as an alternative to hospital medicine, can assist women during pregnancy and child birth. The current study findings indicate that when women experience a good outcome after using herbal medicine, they are likely to repeat its usage in the next pregnancy and viseversa. While some women use herbal medicine to prevent obstetric complications such as postpartum haemorhage, others are reported by caregivers to experience some complications. However, the benefits from self-reported experiences give room for more educative campaigns to reconcile the two different perceptions. Since qualitative findings may inform choice of interventions, this study calls for health professionals to work hand-in-hand with community-based health workers in improving maternal care, thereby preventing any potential obstetric complications aligned to herbal medicine use. Such efforts may contribute to the reduction of maternal mortality in rural Malawi and provision of improved maternal and reproductive health services. Further research can then explore accessibility and community pathway systems for herbal medicine use during pregnancy and labour among the pregnant women in Malawi.

## Supplementary Information

**Additional file 1.**

## Data Availability

The data generated from the current study are not publicly available because we are still writing up from it, but will be available from the corresponding author on reasonable request.
