# Supplementary material for: Herbal medicine use during pregnancy and childbirth: perceptions of women living in Lilongwe rural, Malawi – a qualitative study
Source: BMC Womens Health. 2023 May 4;23:228. doi: 10.1186/s12905-023-02387-z (PMC10158248; doi:10.1186/s12905-023-02387-z)
Supplement: Supplementary file 1 — Additional file 1. [file 12905_2023_2387_MOESM1_ESM.pdf]

Additional file 1: FOCUS GROUP DISCUSSION GUIDE IN ENGLISH

PERCEPTIONS OF WOMEN ON HERBAL MEDICNE USE DURING PREGNANCY AND LABOUR IN LILONGWE

**FGD Cover Page Details**				
Date (month-day-year)				
Catchment Area of (Name setting)				
Recording Code				
FGD duration (in minutes)				
Ask Participant Details	**age**	**parity**		
1.				
2.				
3.				
4.				
5.				
6.				
7.				

Introduction:

Good afternoon/Good morning. My name is Dziwenji Makombe-Mboma, a student studying for a master's degree in Community Health Nursing at Kamuzu College of Nursing. And this is my colleague (*NOTETAKER*). We are very pleased you have agreed to participate in the discussion today. We are here to talk about your perceptions on herbal medicine use during pregnancy and labour. We are very interested to hear your valuable opinions about the taking of herbal medicine use during pregnancy and labour.

• The discussion will provide valuable information that the Ministry of Health can use to improve quality of maternal and new-born health Malawi. The Ministry cannot talk with everyone in Malawi, so your thoughts represent the insights of many women.

• My colleague (*NOTETAKER*) will be taking notes while we talk to help keep track of the conversation. We will also tape record the discussions so that we can make sure to capture the thoughts, opinions, and ideas we hear from you. We understand how important it is that this information is kept private and confidential. Your name (s) will not be recorded.

A. Questions on perceptions on herbal medicine use in pregnancy and labor

I would like to ask you to share with me the perceptions you have regarding herbal medicine use during pregnancy. It could be from your experience, from general knowledge or from what you heard etc. All ideas are welcome.

1. How do you perceive the use of herbal medicine during pregnancy?

Probe 1: If perception is positive tell me more…. Why is that so, can you explain further?

Probe 2: If perception is negative tell me more….Why is that so, can you explain further?

2. How do you perceive the use of herbal medicine during labor?

Probe 1: If perception is positive tell me more…. Why is that so, can you explain further?

Probe 2: If perception is negative tell me more….Why is that so, can you explain further?

B. WOMEN'S BENEFITS AND RISKS FOR HERBAL MEDICINE USE DURING PREGNANCY AND LABOUR

I would like you to share with me the possible benefits and risks that you know, may have experienced, have ever heard in regarding herbal medicine use during pregnancy.

1. What are you told to be the benefits of herbal medicine during pregnancy?
2. What are the actual benefits of using herbal medicine during pregnancy?
3. What are you told to be the benefits of using herbal medicine during labor?
4. What are the actual benefits of using herbal medicine during labor?
5. What are you told are the risks that arise during herbal medicine use in pregnancy?
6. What are the actual risks that arise during herbal medicine use in pregnancy?
7. What are you told are the risks of using herbal medicine during labor
8. What are the actual risks of using herbal medicine during labor?
9. What are the advantages of using herbal medicine during pregnancy/labor over conventional medicine use?

C. DESCRIBE FACTORS THAT INFLUENCE WOMEN TO USE HERBAL MEDICINE DURING PREGNANCY AND LABOUR

1. How do you get to know about herbal medicine?
   Probes:
   - Family
   - Radio
   - Television
   - Traditional Birth Attendants
   - Religion
2. How free are you to refuse the use of herbal medicine during pregnancy/labor?
3. What do you think influences women to use herbal medicine during pregnancy?
   Probes:
   - Family
   - Radio
   - Television

- Bad Obstetric History (Bad Obstetric History)
- Traditional Birth Attendants
- Religion

4. What do you think influences women to use herbal medicine during labor?
   Probes:
   - Family
   - Radio
   - Television
   - Bad Obstetric History (Bad Obstetric History)
   - Traditional Birth Attendants
   - Religion
